# Development of a Specific Monoclonal Antibody to Detect Male Cells Expressing the RPS4Y1 Protein

**DOI:** 10.3390/ijms22042001

**Published:** 2021-02-18

**Authors:** Silvia Spena, Chiara Cordiglieri, Isabella Garagiola, Flora Peyvandi

**Affiliations:** 1 Department of Pathophysiology and Transplantation, Università degli Studi di Milano, 20122 Milan, Italy; silvia.spena@unimi.it; 2 Fondazione IRCCS Ca’ Granda Ospedale Maggiore Policlinico, Angelo Bianchi Bonomi Hemophilia and Thrombosis Center, Fondazione Luigi Villa, 20122 Milan, Italy; isabella_garagiola@yahoo.it; 3 National Institute of Molecular Genetics “Romeo e Enrica Invernizzi”—INGM, 20122 Milan, Italy; cordiglieri@ingm.org

**Keywords:** hemophilia, male cells, male marker, ribosomal protein, RPS4Y1, monoclonal antibody, diagnosis, fetal cells

## Abstract

Hemophilia is an X-linked recessive bleeding disorder. In pregnant women carrier of hemophilia, the fetal sex can be determined by non-invasive analysis of fetal DNA circulating in the maternal blood. However, in case of a male fetus, conventional invasive procedures are required for the diagnosis of hemophilia. Fetal cells, circulating in the maternal bloodstream, are an ideal target for a safe non-invasive prenatal diagnosis. Nevertheless, the small number of cells and the lack of specific fetal markers have been the most limiting factors for their isolation. We aimed to develop monoclonal antibodies (mAbs) against the ribosomal protein RPS4Y1 expressed in male cells. By Western blotting, immunoprecipitation and immunofluorescence analyses performed on cell lysates from male human hepatoma (HepG2) and female human embryonic kidney (HEK293) we developed and characterized a specific monoclonal antibody against the native form of the male RPS4Y1 protein that can distinguish male from female cells. The availability of the RPS4Y1-targeting monoclonal antibody should facilitate the development of novel methods for the reliable isolation of male fetal cells from the maternal blood and their future use for non-invasive prenatal diagnosis of X-linked inherited disease such as hemophilia.

## 1. Introduction

Hemophilia is a congenital bleeding disorder inherited as an X-linked recessive trait. Mutations in the *F8* and *F9* genes, localized on chromosome X at q28 and q27.1, cause the deficiency of the coagulation factor VIII (FVIII) and FIX, respectively. Children with the severe form of hemophilia (i.e., clotting activity of FVIII or FIX less than 1%), suffer from repeated spontaneous bleedings mainly at muscles and joints that result in disabling musculoskeletal damages and chronic arthropathy [1].

In pregnant women carriers of hemophilia, prenatal diagnosis is useful to prepare the family and to plan the delivery, and is usually offered when pregnancy termination would be considered in case of an affected fetus. Since the late 1990s, the prenatal diagnosis in hemophilia has been improved. The current guidelines of the World Federation of Hemophilia state that fetal sex can be determined early (from 7 weeks of gestation) by a non-invasive prenatal test (NIPT) based on analysis of cell free fetal DNA (cffDNA), small (100–150 bp) fragments of DNA released from apoptotic placental cells circulating in the maternal blood [2], through the amplification of Y-linked markers (*SRY*, *DYS14*) [3,4]. However, due to the poor cffDNA quantity and the high maternal DNA contamination (>90%), NIPT cannot be applied for the diagnosis of X-linked inherited diseases, such as hemophilia [5,6,7]. Hence, in case of a male fetus at risk of hemophilia, conventional invasive diagnostic procedures (chorionic villus sampling and amniocentesis) with an associated risk of miscarriage [8] are mandatory to identify in fetuses the maternally inherited genetic defect [9].

As cffDNA, fetal cells (i.e., trophoblastic cells, nucleated red blood cells, granulocytes, lymphocytes, and hematopoietic stem cells) [10] circulate in the maternal blood in number of 4–36 cells/mL [11]. As source of whole fetal genome, circulating fetal cells are an ideal target for NIPT with a potential wider diagnostic range than cffDNA. Nevertheless, the lack of validated and highly specific fetal biomarkers, which enable the unambiguous identification of fetal cells, has been the most limiting factor in all developed strategies for their isolation [12].

To isolate fetal nucleated red blood cells, several antigens highly but not uniquely expressed in erythroid precursors have been tagged such as CD71 [13], glycophorin A [14], γ-hemoglobin [15], and *N*-acetylgalactosamine residues [16]. A specific antibody for a novel surface antigen of fetal erythroblast cells has been recently reported [17]. Similarly, a cocktail of antibodies against endothelial [18] and epithelial markers expressed in circulating endovascular trophoblasts has been developed respectively for their enrichment and staining [19,20]. Moreover, the protein products of *MMP14*, *MCAM*, *KCNQ4*, *CLDN6*, and *F3* genes, expressed in fetal cells, has been proposed as suitable surface markers for fetal cell enrichment [21]. Despite advances in the enrichment technologies based on large size (>15 μm) of trophoblastic cells, such as isolation by size of epithelial tumor/trophoblast filtration and density gradient methods [22,23,24], isolation of fetal cells for clinical implementation remains a technical challenge.

In this scenario, the use of a gender (male) specific biomarker to select circulating fetal cells in carrier women of X-linked diseases would provide for the lack of specific fetal markers. Therefore, the aim of this study was the development of a male-specific monoclonal antibody against the ribosomal protein S4 Y-linked 1 (RPS4Y1), encoded by the Y-linked gene *RPS4Y1*, because specific antibodies against the RPS4Y1 protein are currently unavailable. The RPS4Y1 protein was chosen since previously found in transcriptionally active ribosomes extracted from placenta of a male fetus [25] and expressed in testis and in several somatic tissues of male individuals [26].

## 2. Results

### 2.1. RPS4Y1 Is a Marker of Male Cells

**Figure 1:**
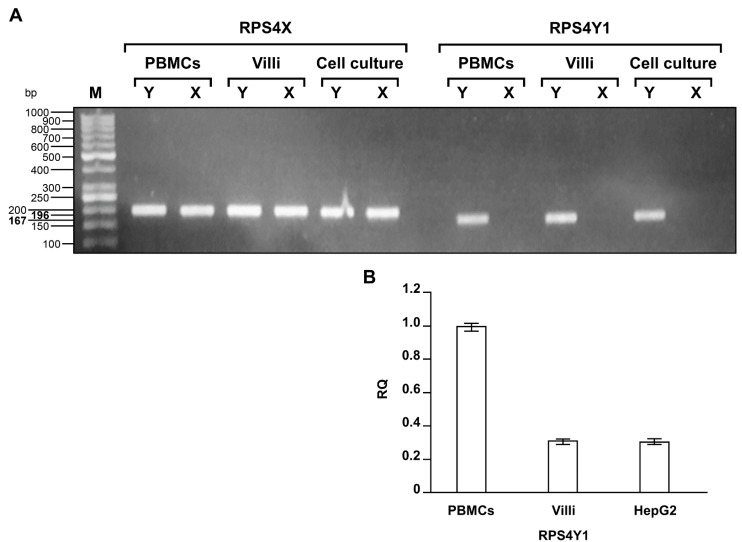
Analysis of RPS4X and RPS4Y1 RT-PCR products. (**A**) Agarose gel electrophoresis showing RPS4X and RPS4Y1 cDNA bands amplified from male (Y) and female (X) peripheral blood mononuclear cells (PBMCs), chorionic villi, male HepG2 and female HEK293 cells. The bands of the GeneRuler 50 bp marker (M) are indicated on the left. Size of RPS4X and RPS4Y1 bands are also reported in bold. (**B**) Bar graph showing the relative quantitation (RQ) of RPS4Y1 transcript in male PBMCs, chorionic villi and HepG2 cells.

The alignment of complementary DNA (cDNA) sequences from the paralogue ribosomal protein S4 X-linked (*RPS4X*) and ribosomal protein S4 Y-linked 1 (*RPS4Y1*) genes (Reference Sequence NM_001007.5 and NM_001008.4, respectively) allowed the design of primers for the specific detection of the two RPS4 isoforms. Reverse transcription polymerase chain reaction (RT-PCR) analysis on total RNA extracted from peripheral blood mononuclear cells (PBMCs) of a male and a female donor showed an expected amplicon of 196 bp amplified by the RPS4X-primer couple in both male and female samples and an expected amplicon of 167 bp amplified by the RPS4Y1-primer couple only in the male sample (Figure 1A). Direct sequencing of RT-PCR products confirmed the specificity of amplification and hence the unique expression of the *RPS4Y1* gene in the male mononuclear cells (data not shown). The same analyses performed on RNA samples from chorionic villi of a male and a female fetuses and from human hepatoma (HepG2) and human embryonic kidney (HEK293) cultured cells, used as unlimited source of biological material and derived respectively from a male and a female [27], confirmed the sex specific expression of the *RPS4Y1* gene (Figure 1A). This result was confirmed by quantitative PCR (qPCR) analysis that showed a reduced but equal expression of RPS4Y1 transcript in male villi and HepG2 cells compared to PBMCs (0.3 vs. 1 relative quantitation) (Figure 1B).

### 2.2. Selection of RPS4Y1-Antigen Peptides

**Figure 2:**
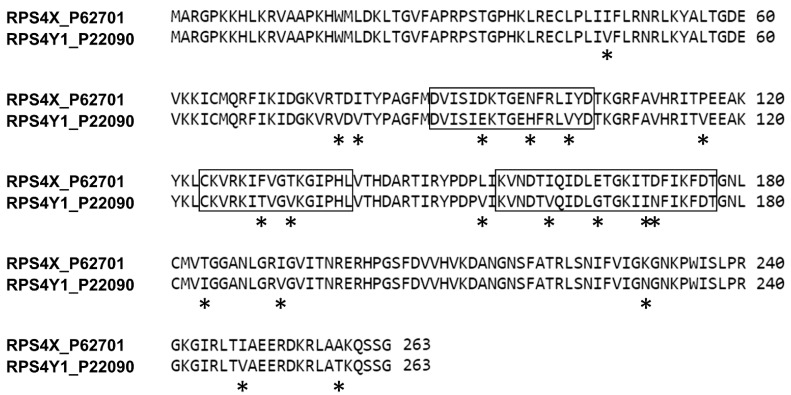
Alignment of RPS4X and RPS4Y1 amino acid sequences. Amino acid difference between the two proteins are indicated by asterisks (∗). Regions selected as antigens for mice immunization are boxed.

The alignment of the reviewed amino acid (aa) sequences of RPS4X and RPS4Y1 (UniProtKB P62701 and P22090, respectively) allowed the identification and localization of 19 aa differences between the RPS4X and the RPS4Y1 homologous proteins (Figure 2). Three small regions (Y1, Y2, Y3 of 17, 16 and 23 aa, respectively) with the highest number of aa specific for the RPS4Y1 protein (2, 3 and 4 respectively) were selected as antigens (Figure 2). To improve immunogenicity, the three small linear peptides selected as antigens were conjugated to keyhole limpet hemocyanin (KLH) carrier protein and used as a pool preparation for mice immunization. After screening for specific binding to the RPS4Y1 pool antigen, four antibody-producing hybridoma clones were selected and corresponding antiRPS4Y1 antibodies have been further analyzed.

### 2.3. AntiRPS4Y1 Antibodies Are Specific for a RPS4Y1-Antigen Peptide and the RPS4Y1 Protein

**Figure 3:**
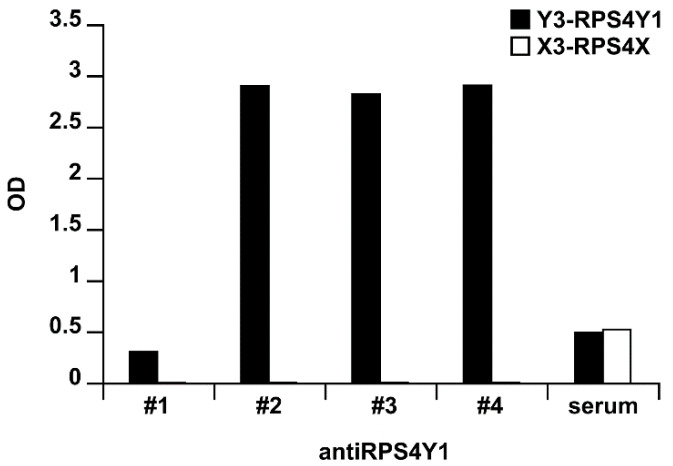
Y3 antigen specificity of monoclonal antibodies. The bar graph showed the optical density (OD; y-axis) from the ELISA assay (one out of three) performed using Y3 (black bar) or X3 (white bar) capture peptides and 2 μg/mL of antiRPS4Y1 antibodies #1, #2, #3, #4 or mouse serum (x-axis).

To evaluate the epitope binding of antiRPS4Y1 antibodies, enzyme-linked immunosorbent assay (ELISA) was performed on individual Y1, Y2, Y3 antigen peptides. No reactivity was evidenced against the Y1 and Y2 peptides (data not shown). By contrast, three antiRPS4Y1 antibodies (#2, #3, #4) showed at each tested concentration (0.5–1–2 µg/mL) similar reactivity for the Y3 peptide (mean optical density, OD: 2.649, 2.775, 2.675 for antibodies #2, #3 and #4, respectively) and no reactivity for the counter-screened X3 peptide (mean OD: 0.012, 0.016, 0.015 for antibodies #2, #3 and #4, respectively), thus suggesting a specific Y3-binding (Figure 3).

**Figure 4:**
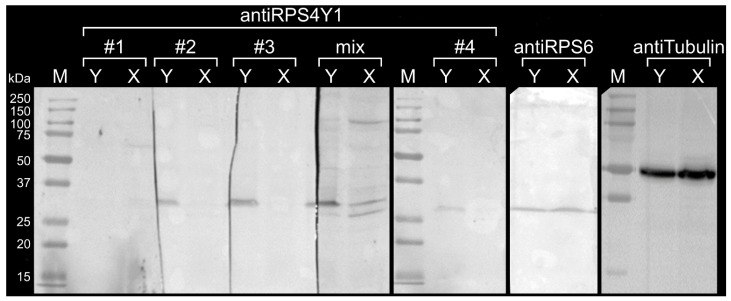
mRPS4Y1 antibody binding to the male RPS4Y1 protein. Representative results of SDS-PAGE and Western blotting (one out of three) performed on cell lysates of male HepG2 (Y) and female HEK293 (X) cells are showed. AntiRPS4Y1 antibodies were tested individually (#1, #2, #3, #4) and in combination (mix). AntiRPS46 and anti-tubulin antibodies were used as loading controls. The bands of the Precision Plus Protein marker (M) are indicated on the left.

The specificity of antiRPS4Y1 antibodies for the entire RPS4Y1 protein was also assessed. Total cell lysates extracted from male HepG2 and female HEK293 cultured cells were separated on sodium dodecyl sulphate-polyacrylamide gel electrophoresis (SDS-PAGE) and Western blotting was performed using antiRPS4Y1 antibodies, either single or in combination. As expected, a single band of approximately 30 kDa, corresponding to the molecular weight of the RPS4Y1 protein, was detected by mRPS4Y1 antibodies #2, #3, #4 in the male sample and not in the female one. This result confirmed the specificity of three monoclonal antibodies (mAbs) for the RPS4Y1 protein and the lack of cross-reactivity with the RPS4X homologous protein expressed both in male and in female cells (Figure 4). The antiRPS4Y1 antibody #1, previously found unresponsive to the RPS4Y1 peptides by the ELISA assay, was used as negative control and confirmed lack of reaction with both male and female samples (Figure 4). By contrast, antibodies against the housekeeping protein tubulin and the ribosomal protein S6 (RPS6), encoded by the *RPS6* gene located in the autosomal chromosome 9 and component of the 40S subunit as RPS4X and RPS4Y1, were used as positive controls. As expected, two single bands corresponding to the molecular weight of tubulin (50 kDa) and RPS6 (28.7 kDa) proteins were evidenced in both male and female cell extracts (Figure 4). The mixture of four antiRPS4Y1 antibodies evidenced a pattern of non-specific bands not only in male but also in female sample without the improvement of the specific signal in the male sample. Among the analyzed antiRPS4Y1 antibodies, the highest specific signal was observed with the antibody #3 (Figure 4).

**Figure 5:**
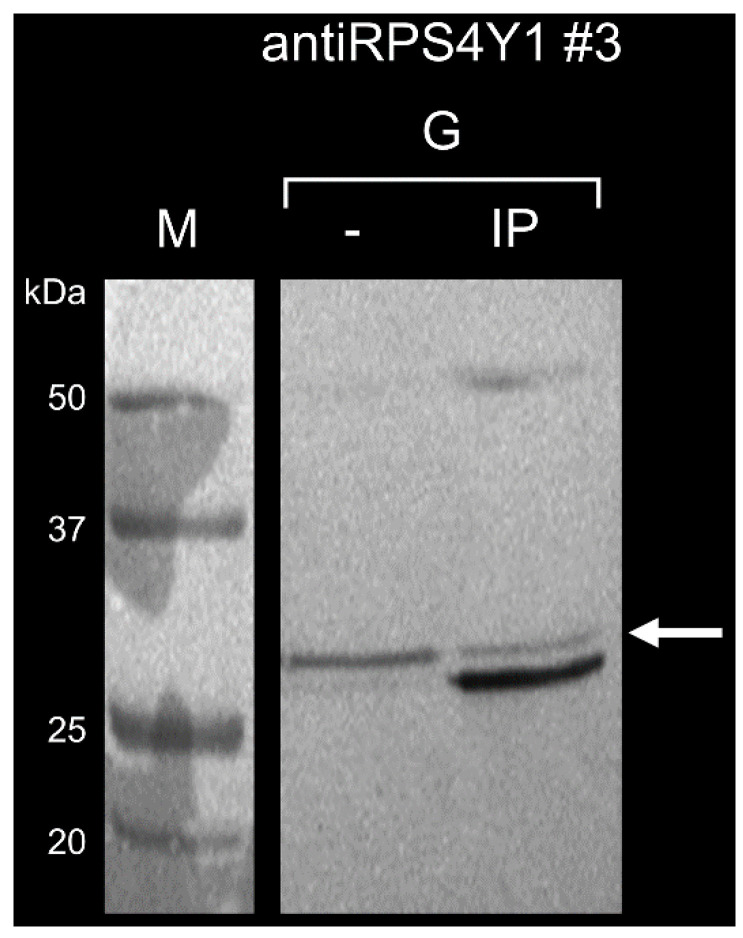
mRPS4Y1#3 antibody binding to the native RPS4Y1 protein. Representative results of immunoprecipitation experiments (one out of three) of RPS4Y1-antibody#3 complex performed with magnetic beads coupled to protein G (G) are showed. Immunoprecipitated proteins (IP) and supernatants (–) were loaded and analyzed by SDS-PAGE and Western blotting. The arrow indicated the RPS4Y1 band.

Immunoprecipitation analysis was further performed to assess the binding ability of antiRPS4Y1 antibody #3 to the native RPS4Y1 protein. Magnetic beads coupled to protein G with high affinity for mouse IgG were used to capture the antibody #3 bounded to the RPS4Y1 protein in HepG2 cell lysate. SDS-PAGE and Western blotting of immunoprecipitated (IP) samples and surnatants (–) showed three bands in IP: a high band and a low band corresponding respectively to 50 kDa-heavy and 25 kDa-light chains of immunoglobulins and an intermediate band corresponding to the 29.4 kDa RPS4Y1 protein (Figure 5). This finding evidenced the ability of the antibody #3 to recognize the RPS4Y1 protein in its native conformation. The presence of the intermediate band in the protein-G supernatant, suggests that not all RPS4Y1 protein is trapped by the antibody.

### 2.4. AntiRPS4Y1 Antibody Is Specific for Male Cells

**Figure 6:**
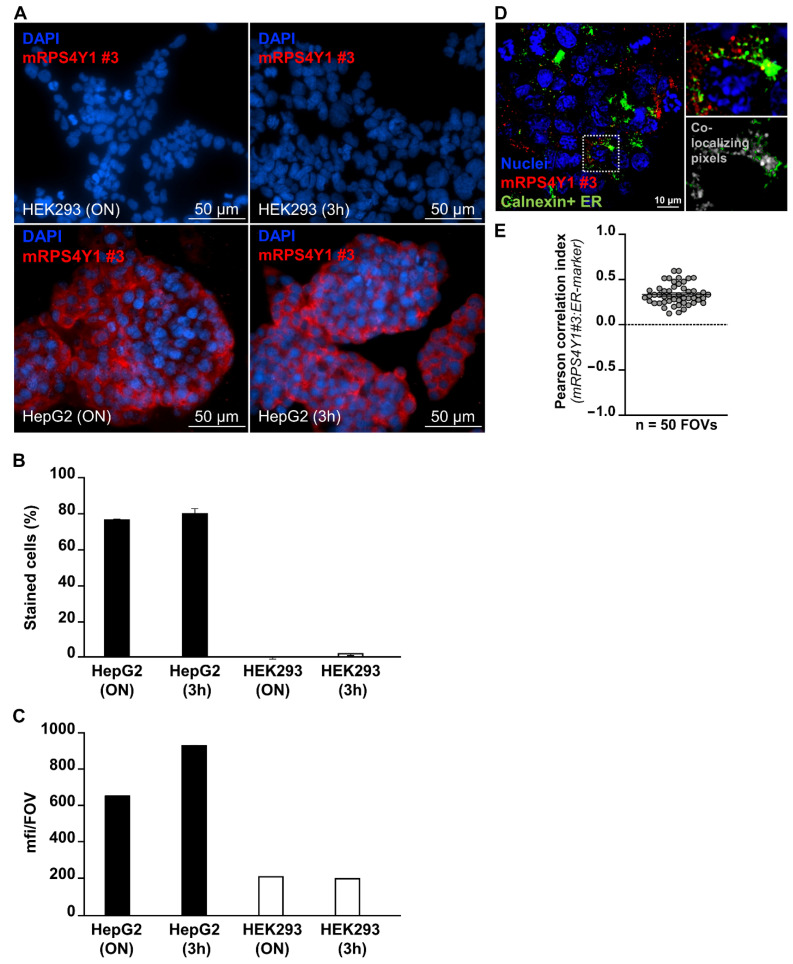
mRPS4Y1#3 antibody specificity for male cells. (**A**) Representative spinning disk confocal microscopy images of mRPS4Y1 antibody #3 staining (red fluorescence) performed over night at 4 °C (ON) or at room temperature for 3 h (3h) on HepG2 and HEK293 cells (lower and upper panels, respectively). (**B**,**C**) Results of the digital imaging analysis of mRPS4Y1 antibody #3 staining reported as the percentage of positively stained cells relative to total cells (**B**) and the mean fluorescence intensity per field of view (mfi/FOV) (**C**). (**D**) Representative spinning disk confocal microscopy at 100× magnification followed by PFS deconvolution via Richardson Lucy algorithm for evaluation of co-localization signals of RPS4Y1 (red fluorescence) and endoplasmic reticulum (ER)-marker calnexin (green fluorescence) on HepG2 cells. Highlighted zoomed-in area in the right upper image showed one single HepG2 cell from the FOV in the left bigger image. Co-localization analysis of signal intensities for each pixel in the FOV is shown in the zoomed in right lower image. Co-localizing pixels with signals from both RPS4Y1 and calnexin are in grey. (**E**) Dot-plot showing the mean Pearson correlation index per FOV resulting from pixel by pixel digital analysis of mRPS4Y1 antibody #3 and ER-marker fluorescent signals performed on 50 FOVs from best-focus deconvolved Z plan at 100× magnification, with a mean number of 30 cells/FOV.

Immunofluorescence analysis was performed to assess the ability of antiRPS4Y1 antibody #3 to detect male cells through the identification of the RPS4Y1 protein. Male HepG2 and female HEK293 cells were both incubated overnight (ON) at 4 °C and at room temperature for 3 h (3 h) with antiRPS4Y1 antibody #3. The almost sole staining of male HepG2 cells compared to female HEK293 cells was observed (76 vs. 0% and 80 vs. 2% for ON and 3 h incubation, respectively), thus suggesting the specific labelling of male cells (Figure 6A,B). Moreover, the observed signal in HepG2 cells was specific since the mean intensity of mRPS4Y1 antibody #3 was higher in male cells than in female ones (mean fluorescent intensity; 659 vs. 215 and 935 vs. 202 for ON and 3 h incubation, respectively) (Figure 6C). Finally, subcellular imaging studies on HepG2 cells were performed. Analysis of RPS4Y1 localization at the level of cellular endoplasmic reticulum (ER) revealed as expected partial co-localization of mRPS4Y1 antibody #3 and ER-marker calnexin (Pearson correlation index 0.4; mean from a total of 1500 analyzed HepG2 cells from n = 50 independent field of view at high-resolution acquisition) (Figure 6D,E).

## 3. Discussion

Current research areas of hemophilia encompass treatment, prevention, and diagnosis of this X-linked inherited coagulation disorder. Recently, improvement on standard replacement treatment, based on infusions of FVIII/FIX, relied on the development of (i) long-lasting replacement factors to reduce the frequency of FVIII/FIX infusions in patients [28], (ii) non-replacement therapies to overcome the problem of the development of neutralizing antibodies [29] and ultimately, (iii) the gene therapy [30]. In the last decades, prevention in hemophilia has been achieved by means of the preimplantation genetic diagnosis that allows the selection of healthy embryos before the in-utero implantation [31]. More recently, prenatal diagnosis in hemophilia has benefited of the development of non-invasive prenatal test for the fetal gender determination. Since the discovery in 1997 of circulating cffDNA in the maternal blood [2], male sex determination has been the first non-invasive test developed for clinical application. Currently, it is adopted in worldwide healthcare systems [32] due to the many advantages relied on safety (a simple maternal blood sample required), high sensitivity and specificity (0.989 and 0.996, respectively) [33], and early (since 7 weeks of gestation) sex determination that has reduced the invasive procedures for the definitive diagnosis by around 50% [34].

The fragmentation and the low amount of cffDNA, that reaches up to 9.7% of total DNA (maternal plus fetal) in the first trimester of pregnancy when the prenatal diagnosis is most useful [35], are crucial issues in the development of non-invasive prenatal diagnosis. Indeed, despite the use of high sensitive detection methods such as digital PCR and next-generation sequencing, commercially available non-invasive prenatal tests based on a sequencing approach to gathering the cffDNA genetic information are for screening purpose and are limited to common aneuploidies (trisomies 21, 18, and 13), sex chromosome abnormalities, and triploidy [36]. Concerning monogenic disorders, many efforts have been made for the development of NIPT. Nevertheless, due to the aforementioned limitations on availability of cffDNA, the best results were obtained for paternally inherited mutations. By contrast, disorders caused by mutant allele inherited from the mother are significantly harder to diagnose because the maternally inherited portion of the fetal DNA is identical to the maternal one. Concerning hemophilia, some non-invasive approaches based on microfluidic dPCR and relative mutation dosage analysis [5,6] and target sequencing and relative haplotype dosage [6,7] have been tested. Despite progressive improvement of methods, none of these have been approved and employed in routine clinical practice.

In this scenario, the gap between the diagnostic sensitivity of invasive procedures and the attractive non-invasive characteristic of cffDNA could be filled by circulating fetal cells (CFCs). Among CFCs, the nucleated red blood cells (nRBC), directly derived from the fetus and with short lifespan, and large placental trophoblasts have been pinpointed for prenatal diagnosis. Due to the extreme rarity of CFCs, ultra-efficient enrichment methodologies have been developed for their isolation. Among them, the microfluidic technologies, such as the frequency-enhanced transferrin receptor antibody-labeled-chip [37] and the NanoVelcro microchip, are the most innovative [24]. Since CFCs recognition occurs through immunological mechanisms, we hypothesized that in pregnancies at risk of X-linked diseases (i.e., pregnancies with a male fetus) the targeting of a male marker expressed in all the CFCs but not in the female (maternal) ones would improve the yield and purity of recovered fetal cells for prenatal diagnosis. To this purpose, we performed a literature search to identify a housekeeping gene on sex chromosome Y expressed in the first trimester of gestation. Among the 70 Y-linked genes, we selected the *RPS4Y1* gene on Yp11.31 as suitable male marker. Indeed, this is one of two genes that exhibits high expression immediately after the embryonic genome activation at the E3 stage (8 cells) and that maintains the expression in the following stages of embryonal development [38]. Moreover, the RPS4Y1 protein has been demonstrated to be expressed in trophoblasts from first-trimester pregnancies [39]. By performing analysis of RPS4Y1 transcript, we confirmed the specific (male) expression of *RPS4Y1* gene on embryonic sample (chorionic villi) and broadened the expression pattern to the liver (HepG2 cells) and blood tissue (PBMCs). These evidences of early and widespread RPS4Y1 expression prompted us to develop a novel monoclonal antibody for a diagnostic application.

The main issue on development of antiRPS4Y1 antibody relied to the high homology between the three members (RPS4X, RPS4Y1, and the ribosomal protein S4 Y-linked 2 (RPS4Y2)) of the eukaryotic S4 family of ribosomal proteins. While the RPS4Y2 paralogue on Yq11.223 is expressed during spermatogenesis [26] and is virtually absent in fetal cells, the RPS4X paralogue on Xq13.1 is functionally equivalent to the RPS4Y1 homologous protein [25]. Located on the exterior (cytoplasmic) side of the ribosomal subunit 40S [40], RPS4X and RPS4Y1 have high sequence identity (approximately 93%) and this high homology makes difficult the production of antibody for the specific detection of the RPS4Y1 protein. To improve the chances of a specific antibody production, most peculiar parts (3) of the RPS4Y1 amino acidic sequence were targeted. Nevertheless, only one of these antigens was immunogenic (155–177 aa). This antigen overlaps a region (155–174 aa) of the RPS4Y1 protein previously used for the production of rabbit antiserum (i.e., polyclonal antibodies) [41], thus suggesting this epitope region as a suitable trigger for the immune response and RPS4Y1 antibody production.

By our immunological analyses, we demonstrated high specificity of the developed monoclonal antibody to both denatured and native RPS4Y1 protein. To the best of our knowledge, this is the first report of a monoclonal antibody against the RPS4Y1 protein. Because our analyses were performed on adult/immortalized instead of fetal cells and an intracellular marker was targeted whose epitope could be partially masked by other proteins/RNAs, further investigations will be necessary to evaluate the sensitivity of our antibody to isolate/identify circulating male fetal cells. In particular, immunofluorescence experiments could be done on (i) chorionic villi that are the main source of circulating fetal cells, (ii) HepG2 and HEK293 cells mixed in vitro to mimic the low number of circulating male fetal cells, and (iii) enriched fetal cells from maternal blood of pregnant women carrier of hemophilia with a male fetus.

Taking advantage from the high uniformity and unlimited quantity of monoclonal antibodies and from the targeting of a gender (male) specific biomarker expressed in any type of male fetal cell, the new antibody here described against the male RPS4Y1 protein would enable the development of strategies for the isolation of circulating male fetal cells with the necessary sensitivity for the development of routine non-invasive prenatal diagnosis of X-linked diseases.

## 4. Materials and Methods

### 4.1. Cell Culture and Mononuclear Cell Isolation

HepG2 and HEK293 cell lines were obtained from the American Type Culture Collection (ATCC, Manassas, VA, USA). HepG2 and HEK293 cells were cultured in Dulbecco’s Modified Eagle’s Medium and Ham’s F12 media (1:1, vol/vol), supplemented with 10% fetal calf serum. Glutamine (1%) and antibiotics (100 IU/mL penicillin and 100 μg/mL streptomycin) were added to both media. Cells were grown at 37 °C in a humidified atmosphere of 5% CO_2_ and 95% air.

PBMCs were isolated from 10 mL of fresh blood samples of healthy individuals using the Ficoll-Paque Premium (GE Healthcare, Chicago, IL, USA). Chorionic villi were obtained by standard clinical procedures. Informed consent was signed prior blood withdrawal and chorionic villus sampling. The study was approved by the Ethics Committee of the Fondazione IRCCS Ca’ Granda, Ospedale Maggiore Policlinico, Milan (520_2017bis, 19 oct 2017) and carried out in accordance with the Declaration of Helsinki.

### 4.2. Protein Extraction and Quantification

Confluent HepG2 and HEK293 cells were washed twice with phosphate buffer saline (PBS) prior the lysis at 4 °C for 15 min with lysis buffer (150 mM NaCl, 50 mM Tris-HCl, 1% Nonidet P-40, 0.1% SDS, pH 8) supplemented with 1% phenylmethylsulfonyl fluoride. Cell lysate was centrifuged at 4000 rpm for 5 min at 4 °C to remove cell debris. Quantification of protein in cell lysate was performed using the DC protein Assay (Bio-Rad, Hercules, CA, USA), according to the manufacturer’s instructions.

### 4.3. RNA Extraction and Reverse Transcription

Total RNA was isolated from PBMCs, chorionic villi, HepG2 and HEK293 cells by using the TRI Reagent^®^ solution (Merck, Darmstadt, Germany). Random nonamers and GoScript™ reverse transcriptase kit with RNAse inhibitor (Promega, Madison, WI, USA) were used to perform first-strand cDNA synthesis starting from 1 μg of total RNA, according to the manufacturer’s instructions.

### 4.4. PCR and qPCR

Primers were designed using the Primer3Plus software (https://primer3plus.com/cgi-bin/dev/primer3plus.cgi, accessed on January 2017). Primers sequences were: RPS4X-F 5′-GCCACTCGACTTTCCAACA-3′, RPS4X-R 5′-CCTGCCACAATATTTTTAATTACG-3′, RPS4Y1-F 5′-TCTTCCGTCGCAGAGTTTCG-3′, RPS4Y-R 5′-TGAGGAAGACGATCAGAGGAA-3′, GAPDH-F 5′-CGACCACTTTGTCAAGCTCAT-3′, GAPDH-R 5′-CCCTGTTGCTGTAGCCAAATT-3′. The specificity of the selected sequences was checked using the Nucleotide BLAST tool (https://blast.ncbi.nlm.nih.gov/Blast.cgi, accessed on January 2017). RPS4X, RPS4Y1 and GAPDH primer couples were used to amplify 50 ng cDNA from each sample using the DreamTaq DNA Polymerase (Thermo Scientific, Waltham, MA, USA). PCR conditions are available upon request. Amplicons were visualized on 2.5% agarose gel. Primer couples were also used to amplify 10 ng of cDNA from each sample using the Fast SYBR Green PCR Master Mix (Applied Biosystems, Waltham, MA, USA). The fluorescence signals were monitored using the StepOnePlus Real-Time PCR system (Applied Biosystems) and StepOne software v2.3 (Applied Biosystems). Triplicates of each sample were analyzed and the relative amount of RPS4Y1 transcript was determined using the 2-∆∆Ct method and the housekeeping *GAPDH* gene as endogenous control.

### 4.5. Sequencing

Direct sequencing of RT-PCR products was performed by the BigDye Terminator Cycle Sequencing Ready Reaction Kit (Applied Biosystems) on an ABI PRISM 3130 Genetic Analyzer (Applied Biosystems). Sequencing analysis was carried out by Sequencing Analysis v3.0 (Applied Biosystems) and sequence alignment was performed using Basic Local Alignment Search Tool (https://blast.ncbi.nlm.nih.gov/Blast.cgi, accessed on February 2017).

### 4.6. Peptide Production and Mice Immunization

Protein alignment of RPS4X and RPS4Y1 (UniProtKB accession numbers P62701 and P22090, respectively) was performed using Clustal Omega tool (https://www.ebi.ac.uk/Tools/msa/clustalo/). KLH and Bovine Serum Albumin (BSA)-coupled peptides corresponding to aa 88-104, 124-139, 155-177 of RPS4X (X1, X2, X3) and RPS4Y1 (Y1, Y2, Y3) were synthesized (Pepscan, Lelystad, The Netherlands). Mice immunization, hybridoma production, hybridoma screening, clone selection, production and purification of antiRPS4Y1 antibodies were performed at PharmAbs (Leuven, Belgium).

### 4.7. Antibody Screening by ELISA

A MaxiSorp 96-well plate (Nunc, Roskilde, Denmark) was coated with 1 µg/mL of each of the BSA-coupled peptides (Y1, Y2, Y3, X1, X2, X3) (100 µL/well in PBS). After incubation overnight at 4 °C, wells were washed with PBS + 0.1% tween 20 and blocked with 200 µL/well of blocking buffer (3% skimmed milk powder in PBS) for 2 h at room temperature (RT). Wells were then washed with PBS + 0.1% tween 20 and incubated with three different concentrations (0.5–1–2 µg/mL) of each antiRPS4Y1 antibody (100 µL/well in PBS + 0.3% skimmed milk powder) for 1 h at RT.

100 µL of serum of immunized mouse (1:1000 in PBS + 0.3% skimmed milk powder) was used as positive control. After washing with PBS + 0.1% tween 20, wells were incubated with 100 µl/well of goat anti-mouse horseradish peroxidase 1:10,000 in PBS + 0.3% skimmed milk for 1 h at room temperature. The plate was developed by adding 160 µL/well of 0.4 mg/mL OPD solution (Merck). Reaction was stopped with 50 µL/well 3M H_2_SO_4_ and absorbance was measured at 490 nm.

### 4.8. Detection of RPS4Y1 Protein by Western Blotting

Cell lysate of HepG2 (60 µg) and HEK293 (120 µg) cells was separated by SDS-PAGE and electrophoretically transferred onto Hybond ECL nitrocellulose membranes (GE Healthcare). The membranes were incubated overnight at room temperature with 2 µg/mL of antiRPS4Y1 monoclonal antibodies. The anti-S6 ribosomal protein monoclonal antibody (MA5-15123; Invitrogen, Waltham, MA, USA) (1:1000) and the anti-alpha tubulin monoclonal antibody (ab11304; Abcam, Cambridge, UK) (1:6000) were used as positive controls. After washing, the membranes were incubated with anti-mouse secondary antibody from sheep conjugated to horseradish peroxidase (Amersham, Buckinghamshire, UK) (1:2000), and visualized by using the Opti-4CN substrate kit (Bio-Rad).

### 4.9. Detection of RPS4Y1 Protein by Immunoprecipitation

20 µg of antiRPS4Y1 monoclonal antibody and 250 µg of cell lysate were incubated overnight at 4 °C. The pre-formed antibody-antigen complex was added to 100 µL of Pure Proteome Protein G magnetic beads (Millipore, Burlington, MA, USA) and incubated for 10 min at room temperature. Denaturing elution was performed and samples were analyzed by Western blotting.

### 4.10. Detection of RPS4Y1 Protein by Immunofluorescence

Male HepG2 and female HEK293 cells were seeded on 8-well chamber-slides (Ibidi, Gräfelfing, Germany) at a density of 5 × 10^4^ cells/well. Cells were grown for 60 h and then fixed with cold 4% Paraformaldehyde for 10 min, permeabilized with 0.2% Triton X-100 for 10 min and blocked for 30 min at RT by using 5% fetal calf serum + 2% goat serum (all from Merck) in PBS. Immunolabelling was performed with 2 µg/mL of mouse primary mAb directed against the ribosomal protein RBS4Y1 or mouse IgG1 kappa isotype control (e-Bioscience, San Diego, CA, USA), followed by secondary antibody labelling conjugated with Alexa Fluor 647 fluorochrome (Molecular Probes, Waltham, MA, USA). For co-immunofluorescence labelling, 2 µg/mL of mouse primary monoclonal antibody directed against ER-marker calnexin (sc-46669; Santa Cruz Biotechnology, Dallas, TX, USA) followed by Alexa Fluor 568-conjugated secondary antibody (Molecular Probes) was used. DAPI (Molecular Probes) was used as nuclear counter labelling. The analysis was reproduced over 6 independently-performed biological experiments, each with independent quadruplicate samples. Images for data quantification were acquired using a fully automated spinning disk confocal microscope equipped with a Nikon Ti widefield inverted microscope (Nikon Instruments, Tokyo, Japan) connected to a CREST X-Light-V2 spinning disk confocal module (CREST-Optics, Rome, Italy), with Andor DU888 EM-gain camera (Andor, Belfast, Northern Ireland), employing a 6-LED excitation source (SpectraAura; LumenCore, Beaverton, OR, USA) and 40× air (NA 0.95) and 60× (Na 1.45) and 100× (NA 1.49) oil objectives (all from Nikon Instruments). A confocal disk with 70-micron pinholes (CREST-Optics) was employed for best Z-sectioning at 200 nm Z-step over 11-micron Z-volumes, correct for adherent cells. Acquisition automation was obtained over an ad-hoc designed pipelines using the JOBS module of NIS-Elements software (Nikon Instruments), created for total sample acquisition and best sample quantification. Automated algorithms were applied for best focal plan detection in each acquired Z-stack and Richardson-Lucy deconvolution algorithm was applied with n = 25 iterations to better resolve the signal PFS and better detect pixel resolution localization. For comparison of acquisition and internal control, selected samples were also acquired using a laser-scanning confocal microscope SP5 (Leica Microsystems, Wetzlar, Germany), employing Las-AF software (Leica Microsystems), with 8-laser lines and 4 photo-multiplicators in sequential modality to avoid any spectral bleed-through, for best co-localization evaluation, using a 63× oil objective (NA 1.40) plus a 3×–9× zoom-in. Digital imaging analysis of mRPS4Y1 positive signal was evaluated in terms of percentage of total cells in each analyzed sample, and in terms of mean fluorescence intensity per field of view (FOV). Quadruplicate biological samples were analyzed for each condition, acquiring 9 FOVs for each sample at 40× and 60× magnification. A total number of 10,946 HEK293 cells and 11,304 HepG2 cells were analyzed via specifically-scripted general analysis pipeline of object segmentation and image measurement, using NIS-Elements v.5.11 (Lim-Nikon). The signal-pixel correlation analysis, for mRPS4Y1 sub-cellular co-localization studies, was performed over 50 independently acquired FOVs for each tested condition at 100x magnification. The focused Z-plans were deconvolved using the Richardson-Lucy algorithm and used for co-localization analysis, which was performed both via NIS-Elements and via Fiji Coloc 2 plugin, for internal analytical control. Co-localization was evaluated using the Pearson correlation index. Values, ranging from −1 (no correlation) to +1 (complete correlation) were plotted and the statistical analysis was performed using Kolmogorov test to normal distribution evaluation, followed by 1-way ANOVA with Bonferroni post-hoc comparisons using Prism Graph-Pad V.8 (GraphPad Software, San Diego, CA, USA).

## Data Availability

Data sharing not applicable.
